# A Clinical Decision‐Making Tool to Identify Red Flags for Remote Cognitive Assessment: An Expert Consensus Study from the Canadian Consortium on Neurodegeneration in Aging

**DOI:** 10.1002/alz.088902

**Published:** 2025-01-09

**Authors:** Nathan Friedman, Inbal Itzhak, Philippe Desmarais, John D Fisk, Linda Lee, Alexandre Henri‐Bhargava, Jacqueline Pettersen, Paula M McLaughlin, Zahinoor Ismail, Vladimir Khanassov, Morris Freedman, Howard Chertkow, Richard Camicioli, Megan E O'Connell, Maiya R. Geddes

**Affiliations:** ^1^ McGill University, Montreal, QC Canada; ^2^ CCNA, Lady Davis Institute, Jewish General Hospital, Montreal, QC Canada; ^3^ Centre hospitalier de l'Université de Montréal, Montréal, QC Canada; ^4^ Dalhousie University, Halifax, NS Canada; ^5^ MINT Memory Clinic, Kitchener, ON Canada; ^6^ University of Victoria, Victoria, BC Canada; ^7^ University of British Columbia, Prince George, BC Canada; ^8^ Queens University, Kingston, ON Canada; ^9^ Hotchkiss Brain Institute, University of Calgary, Calgary, AB Canada; ^10^ Department of Medicine, University of Toronto, Toronto, ON Canada; ^11^ University of Toronto, Toronto, ON Canada; ^12^ Baycrest and Rotman Research Institute, Toronto, ON Canada; ^13^ University of Alberta, Edmonton, AB Canada; ^14^ University of Saskatchewan, Saskatoon, SK Canada; ^15^ Montreal Neurological Institute‐Hospital (The Neuro), Montreal, QC Canada

## Abstract

**Background:**

Remote diagnostic assessment of cognitively impaired individuals offers numerous potential benefits including increased access to care. However, remote cognitive and behavioral assessment also has limitations, and may not be appropriate for certain patients. Currently, evidence‐based guidance on virtual assessment readiness is lacking. Our goal was to develop a clinical decision‐making tool that outlines an approach to determining a patient’s suitability for undergoing remote cognitive and behavioral diagnostic assessment by identifying ‘red flags’ for remote assessment. To address this goal, a multidisciplinary workgroup was convened under the auspices of the Canadian Consortium on Neurodegeneration in Aging (CCNA). This workgroup was composed of experts in remote assessment and included behavioral neurologists, neuropsychiatrists, neuropsychologists, social workers, geriatricians, persons with lived experience and family medicine specialists.

**Methods:**

The Delphi process is an iterative, systematic, group consensus method, used here to determine the features of the patient, caregiver, clinician and context/situation, or ‘red flags’, indicating that a remote cognitive diagnostic assessment should be avoided. The process consisted of anonymized data collection in three rounds among the multidisciplinary expert workgroup, culminating in two rounds of iterative scoring of potential red flags based on three quality indicators that assessed a potential red flag’s effectiveness, reproducibility, and efficiency. Red flags that received an overall mean score above the pre‐determined consensus threshold on the final round were included in the final clinical decision‐making tool.

**Result:**

In the first round, 11 respondents, with an average of 12.4 years of clinical experience, generated 67 unique potential red flags. In the second and third rounds, 8 and 9 respondents, respectively, scored the flags on the three quality indicators. Applying consensus criteria yielded 14 red flags that achieved consensus.

**Conclusion:**

To enhance the translation and implementation of these findings, we developed a clinical decision‐making tool and infographic describing the final set of red flags in collaboration with the CCNA knowledge translation team. This infographic is designed to help clinicians determine a patient’s readiness to undergo remote cognitive assessment. This study directly impacts the clinical care of cognitively impaired individuals by providing clinical decision‐making guidance on a patient’s suitability for remote neurobehavioral assessment.

